# Effect of Different Light Quality and Photoperiod on Mycelium and Fruiting Body Growth of *Tricholoma giganteum*

**DOI:** 10.3390/life16010039

**Published:** 2025-12-26

**Authors:** Qingqing Luo, Meirong Zhan, Shengze Yan, Ting Xie, Xianxin Huang, Ruijuan Wang, Huan Lu, Shengyou Wang, Juanjuan Lin

**Affiliations:** 1 Fujian Provincial Key Laboratory of Crop Genetic Improvement and Innovative Utilization, Institute of Edible Fungi, Sanming Academy of Agricultural Sciences, Sanming 365509, China; luoqingqing6@126.com (Q.L.); zhmrnhkl@163.com (M.Z.); yanshengze1102025@163.com (S.Y.); 19376776419@163.com (T.X.); hxianxin96@163.com (X.H.); 2 Key Laboratory of Edible Fungi Resources Utilization in South China, National Edible Fungi Germplasm Resource Bank, Shanghai Key Laboratory of Agricultural Genetics and Breeding, Institute of Edible Fungi, Shanghai Academy of Agricultural Sciences, National Edible Fungi Engineering Technology Research Center, Shanghai 201403, China; wangruijuan@saas.sh.cn (R.W.); luhuan@saas.sh.cn (H.L.); 3 College of Life Sciences, Fujian Normal University, Fuzhou 350117, China

**Keywords:** antioxidant activity, LED lighting, photomorphogenesis, nutrient content, *Tricholoma giganteum*

## Abstract

Light is a crucial environmental regulator for *Tricholoma giganteum* (*T. giganteum*). This study investigated the effects of light quality and photoperiod on its growth, physiology, and nutritional composition. During the mycelial stage, blue light (BL) exposure for 5 d promoted the highest growth rate (0.74 mm d^−1^, 45% higher than dark control, *p* < 0.05). Red light (RL) enhanced antioxidant capacity, elevating superoxide dismutase (SOD) activity to 240.20 U·mL^−1^ (after 5 d) and DPPH radical-scavenging activity to 276.11% (after 3 d). Ultraviolet (UV) suppressed polyphenol oxidase (PPO) activity. BL also increased mycelial polysaccharide content (6.45 g·100 g^−1^). In the fruiting stage, green light (GL) improved agronomic traits and first-grade yield (3.75 kg), while also promoting the accumulation of glutamate (4.39 g·100 g^−1^), a key umami compound. Further photoperiod optimization revealed that 4 h of daily GL exposure shortened the fruiting cycle, achieved the highest biological efficiency (98.4%), and maximized both polysaccharide (38.17 g·100 g^−1^) and glutamate contents (5.70 g·100 g^−1^). These results recommend a two-stage lighting protocol: BL for mycelial growth and a 4 h daily GL for fruiting, providing a scientific basis for the industrial cultivation of *T. giganteum*.

## 1. Introduction

*Tricholoma giganteum* (*T. giganteum*) is a highly valued edible mushroom in East Asia, prized not only for its distinctive flavor and large fruiting body but also for its rich nutritional profile and purported medicinal properties, including antioxidant and immunomodulatory effects [1]. Its cultivation during high-temperature seasons fills a market gap, showing great commercial potential in subtropical regions [2]. However, the industry still relies on empirical practices, particularly regarding environmental control [3].

Among the different environmental factors, light is the most important factor that affects fungi gene expression, enzyme activity, growth, development, and nutritional composition [4,5,6]. Light is a pivotal environmental cue for fungi, governing morphogenesis, phototropism, and metabolic pathways [7]. For instance, dark conditions may cause the fruiting bodies to deform, the stipes to elongate, and the caps to change color [2,8,9]. The blue light (BL) stimulates fruiting body development and enhances cordycepin content in *Cordyceps militaris* [10], and specific intensities of BL significantly improve fruiting body formation in *Pleurotus eryngii* [11]. Furthermore, green light (GL) promotes the growth and nutrient synthesis of *Ganoderma lucidum* by enhancing its extracellular enzyme activity. However, red light (RL) inhibits the differentiation of its fruiting bodies [12]. Research on *Tremella fuciformis* also shows that white light (WL) increases individual basidiocarp weight and polysaccharide content, whereas yellow light favors protein accumulation [13].

Beyond triggering fruiting body formation, light quality profoundly influences fungal physiology [14]. Specifically, different wavelengths can modulate the activity of antioxidant enzyme systems such as superoxide dismutase (SOD), catalase (CAT), and peroxidase (POD), which are crucial for stress tolerance and delaying postharvest browning [7,15,16,17,18]. BL can significantly increase the activity of antioxidant enzymes and enhance the antioxidant defense system in organisms [19]. The ethanol extract from the fruit body and mycelium of P. ostreatus, exposed to ultraviolet-B (UV-B), shows a high content of vitamin D2, suggesting that both the fruit body and mycelium could serve as potential antioxidants and be developed into novel dietary supplements [20,21,22]. Moreover, light regulates the biosynthesis of valuable secondary metabolites, such as polysaccharides, proteins, polyphenols, and ergosterol, directly impacting the nutritional and medicinal quality of edible mushrooms [23,24,25].

While the photobiology of model fungi like *Neurospora crassa* is well-studied, applied research on edible species has yielded species-specific results. For instance, BL promotes cordycepin synthesis in *Cordyceps militaris*, while GL enhances the yield of *Ganoderma lucidum* [10,12]. However, a systematic understanding of light responses in *T. giganteum* across its lifecycle is lacking. Existing studies often focus on a single growth stage or a narrow light spectrum, failing to link light quality to both developmental and physiological outcomes. To address this gap, this study aimed to systematically investigate the effects of specific light qualities (BL, GL, WL, UV) and photoperiod on *T. giganteum*.

Therefore, the primary objective of this study was to systematically investigate the effects of specific light qualities (BL, GL, WL, UV) and an optimized photoperiod on *T. giganteum* across its lifecycle. We hypothesized that BL would optimally promote mycelial growth due to known fungal BL photoreceptors, and GL would enhance fruiting body yield and nutritional quality by modulating carbohydrate metabolism and antioxidant systems. This work aims to provide a scientific foundation for precise light management in the industrial cultivation of *T. giganteum*.

## 2. Materials and Methods

### 2.1. Materials

#### 2.1.1. Fungal Strains

The strain of ‘Langkou *T. giganteum*’ was used in this study. It was originally isolated from a wild basidiocarp in Sanming, Fujian Province, China, and is preserved in the Culture Collection of the Edible Fungi Research Institute, Sanming Academy of Agricultural Sciences, under accession number K-1.

#### 2.1.2. Cultivation Medium

The mycelial cultivation medium, potato dextrose agar (PDA), contained the following per liter: 200 g of potato extract, 20 g of glucose, and 20 g of agar. The pH was natural [1]. The fruiting body cultivation substrate comprised (dry weight basis): 35% edible fungus cultivation waste, 48% corn cob, 15% wheat bran, and 2% lime. Moisture content was adjusted to approximately 65% after mixing thoroughly.

#### 2.1.3. Light Treatment Set Up

Custom LED panels (Shenzhen, China) were used to provide monochromatic light. They emitted red (630 ± 10 nm), blue (450 ± 10 nm), green (525 ± 10 nm), white (full spectrum), and UV (365 nm) wavelengths. For mycelial stage experiments, Petri dishes were placed 30 cm beneath the light source. The light intensity (Photosynthetic Photon Flux Density, PPFD) at the culture surface was measured using a quantum sensor (LI-250A, LI-COR, Lincoln, NE, USA) and adjusted to 50 μmol m^−2^ s^−1^ for all light quality treatments. Dark conditions (CK for mycelium) were achieved by wrapping cultures in aluminum foil.

For fruiting body stage experiments, ‘Langkou *T. giganteum*’ cultivation bags were placed in an artificial intelligence mushroom shed. Light sources were positioned 50 cm above the substrate surface, providing a uniform light intensity of 150 lux (approximately 30 μmol m^−2^ s^−1^ PPFD) as measured by a lux meter (LX-1010B, Xinbaorui, Xi’an, China). To prevent light interference between treatments, different light sources were physically separated by black shading cloth.

### 2.2. Measurement of Growth Parameters

Mycelial plugs (5 mm diameter) of *T. giganteum* were inoculated at the center of Petri dishes and incubated at 28 °C under various light conditions: RL, BL, GL, UV, WL, and darkness as the control (CK). Each light quality treatment group was exposed for 3, 5, and 10 d. Each treatment comprised six plates, with three biological replicates. Colony diameter was measured using the cross method, starting from the third day after inoculation and subsequently every three days. The mean diameter was calculated from two perpendicular measurements [1]. The mycelial growth rate (mm·d^−1^) was determined using the following formula: 1/2 × colony diameter/growth time. Mycelial density was visually assessed and categorized as follows: very thin (+), thin (++), thick (+++), and very thick (++++).

At harvest, key agronomic traits of the fruiting bodies—including stipe length, stipe diameter, pileus diameter, and pileus thickness—were measured using a digital vernier caliper (Mitutoyo Corporation, Kawasaki, Kanagawa, Japan; precision: 0.01 mm). The fresh weight of individual mushrooms was also recorded. Based on the performance under different light qualities, GL was selected as the most promising for fruiting body development. To optimize its application, we further investigated the effect of photoperiod. A gradient of daily GL exposure durations (2, 4, 8, 12, and 24 h) was tested, with continuous WL serving as the control treatment.

### 2.3. Biochemical Analyses

#### 2.3.1. Antioxidant and Enzyme Activity Assays

All enzymatic assays were performed on fresh liquid nitrogen tissue homogenates.

##### Superoxide Dismutase (SOD)

SOD activity was assayed using a kit (Solarbio, Beijing, China) based on the inhibition of nitroblue tetrazolium (NBT) photoreduction [24]. One unit (U) of SOD activity was defined as the amount of enzyme required to inhibit the reduction of nitroblue tetrazolium (NBT) by 50% per minute per milligram of protein (U·mg^−1^ protein).

##### Polyphenol Oxidase (PPO)

PPO activity was determined by monitoring the oxidation of catechol at 420 nm [26]. Activity was expressed as the change in absorbance per minute per mg protein (ΔA420/min/mg prot).

##### DPPH

DPPH Radical Scavenging Activity was measured according to the method of Tian et al. [20] with modifications. Briefly, 0.1 mL sample extract was mixed with 3.9 mL of 0.1 mM DPPH methanol solution. After 30 min incubation in darkness, absorbance was measured at 515 nm. The scavenging activity was calculated as: Scavenging activity (%) = [(Control − Sample)/Control] × 100%. Results are expressed as % scavenging activity.

#### 2.3.2. Nutrient Component Analysis

##### Polysaccharide

Samples of mycelium and fruiting bodies from different light treatments were dried at 60 °C to constant weight, ground, and passed through a 60-mesh sieve. Polysaccharides were then extracted from the powdered samples using the phenol-sulfuric acid method [27]. Using glucose as the reference substance, the glucose concentration is set as the abscissa (X), and the absorbance value as the ordinate (Y). The standard curve is calculated using Formula (1), and the polysaccharide content is determined based on the standard curve.

(1) Y = 8.7905X + 0.02068 (R^2^ = 0.998)

##### Protein

The protein concentration of the samples was determined using a bicinchoninic acid (BCA) assay kit (Solarbio, China) according to the manufacturer’s instructions [28]. Briefly, bovine serum albumin (BSA) was used as the standard. Each standard or appropriately diluted sample (10 μL) was mixed with 200 μL of the BCA working reagent in a 96-well microplate. The plate was incubated at 37 °C for 30 min. The absorbance was measured at 562 nm using a SYNERGY HT multi-mode microplate reader (BioTek Instruments, Inc., Winooski, VT, USA). The protein concentration of each sample was calculated by interpolating its absorbance against the standard curve. The standard curve is calculated using Formula (2); all measurements were performed in triplicate.

(2) Y = 1.2676X + 0.1134 (R^2^ = 0.996)

##### Glutamate

The glutamate concentration was determined using an M-100 Biosensor Analyzer [29,30]. After centrifugation and dilution, a 25 μL aliquot of the supernatant was injected. The concentration was directly calculated by the instrument based on a pre-established standard curve, utilizing the specific electrocatalytic signal generated from the immobilized L-glutamate oxidase membrane. Each sample was analyzed in triplicate.

### 2.4. Statistical Analysis

All experiments were conducted following a completely randomized design with biological replicates (at least three independent replicates per treatment). Data were analyzed using IBM SPSS Statistics (version 27.0; IBM Corp., Armonk, NY, USA; https://www.ibm.com/products/spss-statistics, accessed on 22 August 2025). One-way or two-way analysis of variance (ANOVA) was performed, followed by Tukey’s honestly significant difference (HSD) post hoc test for mean separation at a significance level of *p* < 0.05. Data are presented as mean ± standard deviation (SD).

## 3. Results

### 3.1. Agronomic Traits Analysis

#### 3.1.1. Effects of Different Light Qualities on Mycelial Growth of *T. giganteum*

The results of the study demonstrated a significant influence of different light qualities on both the growth rate and morphology of *T. giganteum* mycelia (Table 1). Under short-term illumination (3 d), mycelial growth significantly increased under both BL and GL treatments compared to the control (CK). As the duration of illumination increased, the mycelial growth rate under BL treatment peaked at 5 days, accompanied by optimal morphology. However, an extension of the light exposure period resulted in a substantial decrease in the growth rate. In contrast, UV treatment markedly suppressed mycelial growth across all tested exposure durations.

#### 3.1.2. Effects of Different Light Qualities on the Growth and Development of *T. giganteum* Fruiting Bodies

**Figure 1:**
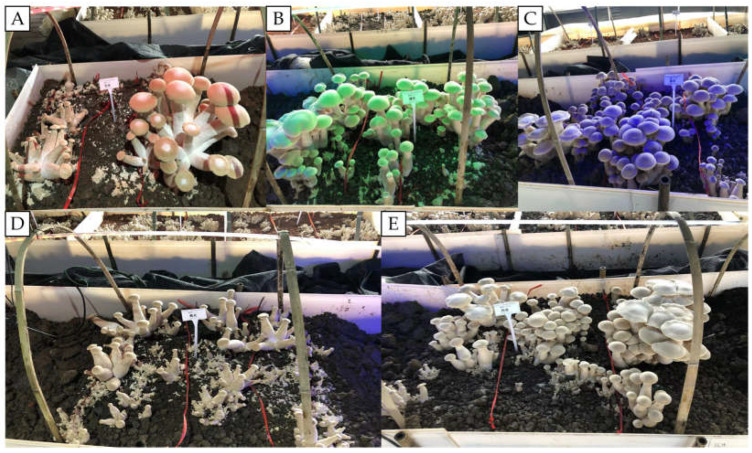
Growth and development of fruiting bodies of *T. giganteum* under different light conditions. (**A**) RL; (**B**) GL; (**C**) BL; (**D**) YL; (**E**) WL.

The study found that different light qualities significantly affected the growth and development of *T. giganteum* fruiting bodies. As demonstrated in Figure 1, the application of RL treatment resulted in the production of larger fruiting bodies; however, their internal tissue exhibited a hollow, spongy structure, which consequently led to poor commercial quality. YL treatment resulted in a reduction in yield and a decrease in the size of individual fruiting bodies. Conversely, both BL and GL treatments resulted in increased yields. It is noteworthy that the application of GL resulted in the production of fruiting bodies that exhibited thicker stipes and a more compact texture, thereby demonstrating superior agronomic characteristics.

Regarding the fruiting process, GL treatment significantly shortened the time required for both primordium formation and fruiting, advancing these stages by 8 d compared to the conventional WL (CK), which demonstrates a notable growth-promoting effect. In terms of yield, BL treatment resulted in the highest average yield per bag (0.335 kg) and biological efficiency (67%), while GL treatment followed closely with an average yield per bag of 0.322 kg and a biological efficiency of 66.4%. However, the yield of first-level mushrooms under GL reached 3.751 kg, representing the highest among all treatments (Table 2). Further analysis of agronomic traits revealed that RL treatment produced the longest fruiting bodies (19.69 mm), followed by GL (16.43 mm), whereas BL treatment performed best in terms of pileus thickness and stipe diameter. Regarding average single mushroom weight, GL treatment yielded the heaviest fruiting bodies (105.926 g), slightly higher than those under RL (103.564 g). Overall, both GL and BL demonstrated significant advantages in promoting fruiting, increasing yield, and optimizing agronomic traits (Table 3).

#### 3.1.3. Effects of Different Durations of GL Exposure on the Growth and Development of *T. giganteum* Fruiting Bodies

**Figure 2:**
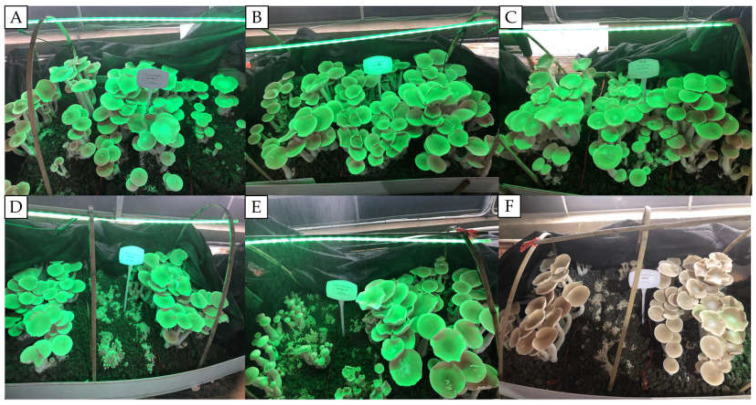
Growth and development of fruiting bodies of *T. giganteum* under different light durations of GL. (**A**) 2 h; (**B**) 4 h; (**C**) 8 h; (**D**) 12 h; (**E**) 24 h; (**F**) 0 h (white light exposure 24 h).

The duration of GL exposure significantly influenced the growth and development of *T. giganteum* fruiting bodies. As shown in Figure 2, the treatment with 4 h of daily GL exposure demonstrated optimal performance in both yield and morphology, producing fruiting bodies with thick stipes and compact texture; whereas continuous 24 h GL exposure resulted in reduced yield and promoted premature cap opening, thereby diminishing commercial value (Table 4). Regarding the fruiting process and yield (Table 4), 4 h of GL exposure significantly shortened both primordium formation and fruiting time, advancing these stages by 9 d compared to the conventional WL (CK). This treatment also achieved the highest average yield per bag (0.492 kg/bag) and biological efficiency (98.4%). Further analysis indicated that yield progressively increased as the light duration extended from 2 h to 4 h; however, further extension of light exposure led to a decline in yield, with both the yield per bag and biological efficiency reaching their lowest values (0.388 kg/bag and 77.4%, respectively) under continuous 24 h illumination. Agronomic trait analysis further supported these trends (Table 5). The 4 h GL treatment showed optimal performance in fruiting body length (24.608 cm), pileus thickness (25.016 mm), pileus diameter (12.984 cm), and average single weight (102.4 g). All agronomic traits exhibited a declining trend with prolonged light exposure, indicating that moderate GL irradiation promotes morphogenesis and substance accumulation, while excessive exposure exerts inhibitory effects.

### 3.2. Physiological Enzyme Activity and Antioxidant Analysis

#### 3.2.1. Effects of Different Light Qualities on the Physiological Enzyme Activities and Antioxidant of *T. giganteum* Mycelium

Different light quality treatments significantly affected the physiological enzyme activities in the mycelia of *T. giganteum*. The activity of PPO reached its peak (135.54 U·mL^−1^) after 3 d of UV treatment, but decreased significantly to 62.76 U·mL^−1^ when the treatment was extended to 10 d. The DPPH radical scavenging capacity and SOD activity exhibited similar trends under RL treatment. Both reached their highest levels after 3 d (DPPH) and 5 d (SOD) of RL exposure, with values of 276.11% and 240.20 U·mL^−1^, respectively. However, when the RL treatment was prolonged to 10 d, both the DPPH scavenging capacity and SOD activity significantly declined, dropping to 122.43% and 144.21 U·mL^−1^, respectively. These results indicate that short-term RL irradiation enhances the antioxidant capacity of the mycelia, whereas prolonged exposure exerts inhibitory effects (Table 6).

#### 3.2.2. Effects of Different Light Qualities on the Physiological Enzyme Activities and Antioxidant of *T. giganteum* Fruiting Bodies

Different light qualities significantly regulated the physiological and antioxidant activities in the fruiting bodies of *T. giganteum* (Table 7). Specifically, the activity of PPO was highest under YL treatment, reaching 136.39 U·mL^−1^; BL treatment was most conducive to enhancing SOD activity, which reached 186.06 U·mL^−1^; while the DPPH radical scavenging capacity was strongest under GL treatment, attaining 148.01%. These results indicate that both BL and GL exhibit positive effects on enhancing the antioxidant enzyme system in the fruiting bodies.

#### 3.2.3. Effects of Different Durations of GL Exposure on the Physiological Enzyme Activities and Antioxidants of *T. giganteum* Fruiting Bodies

The duration of GL exposure significantly influenced the physiological and antioxidant activities in the fruiting bodies of *T. giganteum*, with SOD, PPO, and DPPH scavenging capacity exhibiting distinct response patterns to the photoperiod (Table 8). SOD activity demonstrated an initial increase followed by a subsequent decrease as the duration of light exposure increased. The activity reached a maximum of 176.06 U·mL^−1^ under the 8 h treatment and decreased to its lowest level under continuous 24 h illumination. Conversely, DPPH activity exhibited a continuous increase with prolonged light exposure, reaching its maximum level of 139.01 U·mL^−1^ under the 24 h treatment. In a similar manner, the PPO activity exhibited an initial increase followed by a subsequent decrease, with its peak value (134.31 U·mL^−1^) observed in the 8 h treatment group before gradually declining.

### 3.3. Nutrient Content Analysis

#### 3.3.1. Effects of Different Light Qualities on Nutrient Content of *T. giganteum* Mycelium

The results of the study demonstrated that both light quality and exposure duration significantly influenced the nutrient accumulation in the mycelia of *T. giganteum* (Table 9). The contents of protein and polysaccharides exhibited an increasing trend with prolonged illumination time. The protein content attained its maximum level (3.55 g·100 g^−1^) following 10 d of cultivation under dark conditions (CK), while it exhibited its minimum level (0.33 g·100 g^−1^) after 3 d of UV irradiation. The polysaccharide content reached its maximum level (6.45 g·100 g^−1^) after 10 days of BL treatment and exhibited its minimum level (0.39 g·100 g^−1^) under short-term UV exposure. Furthermore, the glutamate content was highest (1.472 g·100 g^−1^) after three days of GL treatment and lowest (0.904 g·100 g^−1^) under UV, indicating that GL promotes the synthesis of umami-related amino acids in the mycelia.

#### 3.3.2. Effects of Different Light Qualities on the Nutrient Content of *T. giganteum* Fruiting Bodies

The application of diverse light quality treatments exerted a substantial influence on the regulation of nutrient accumulation in the fruiting bodies of *T. giganteum* (Table 10). Among the treatments, the protein content was highest under YL treatment (35.924 g·100 g^−1^), while BL treatment resulted in the lowest levels of both protein and polysaccharides (32.538 g·100 g^−1^ and 18.509 g·100 g^−1^, respectively). Conversely, GL treatment exhibited remarkable efficacy in enhancing the accumulation of active components and flavor substances, demonstrating the highest polysaccharide content (31.007 g·100 g^−1^) and glutamate content (4.390 g·100 g^−1^) among all treatments. In contrast, the RL treatment resulted in the lowest glutamate content (3.152 g·100 g^−1^), indicating a possible inhibitory effect on the formation of umami substances.

#### 3.3.3. Effects of Different Durations of GL Exposure on Nutrient Content of *T. giganteum* Fruiting Bodies

The duration of GL exposure significantly influenced the nutrient accumulation in the fruiting bodies of *T. giganteum* (Table 11). As the exposure time increased from 2 to 4 h, the contents of both protein and polysaccharides significantly increased, reaching their peak values (35.798 g·100 g^−1^ and 38.169 g·100 g^−1^, respectively) under the 4 h treatment. Conversely, the 8 h treatment resulted in the highest glutamate content (5.752 g·100 g^−1^). However, when the light exposure was further extended, the levels of all three nutritional components exhibited a downward trend, indicating that moderate light duration promotes the synthesis and accumulation of nutrients, whereas excessive exposure may exert inhibitory effects.

## 4. Discussion

This study provides the first comprehensive analysis of light quality effects across the lifecycle of *T. giganteum*, revealing stage-specific photomorphogenic responses with direct implications for industrial cultivation. The findings that BL optimizes mycelial growth, while GL enhances fruiting body yield and quality (Table 1, Table 2 and Table 3), support our initial hypotheses and suggest distinct regulatory mechanisms at play during different developmental phases. These results demonstrate the potential of precise light regulation to advance sustainable food production, which aims to provide nutritious food with low environmental impact and cost [31].

The promotion of mycelial growth by BL, particularly the peak growth rate observed after 5 days of exposure (Table 1), aligns with the conserved function of fungal BL photoreceptors, such as the White-Collar Complex (WCC) [32]. In *Flammulina filiformis*, BL perception by WCC upregulates genes involved in cell wall remodeling and nutrient transport, leading to accelerated hyphal extension [33]. The decline in growth rate after prolonged BL exposure may indicate a feedback inhibition mechanism or light-induced oxidative stress, a balance that requires precise timing in application. Conversely, the strong inhibition by UV light is likely due to DNA damage, consistent with its role as a genotoxic stressor across biological systems.

At the fruiting stage, the superiority of GL in improving yield and agronomic traits (e.g., pileus diameter, average weight) is particularly noteworthy (Table 2 and Table 3). This contrasts with some studies where GL had minimal effects but resonates with findings in *Cordyceps militaris* and *Ganoderma lucidum* [10,12]. We propose that GL may enhance the activity of carbohydrate-active enzymes like amylases, as suggested for other fungi, improving the mobilization of carbon sources from the lignocellulosic substrate (corn cob), thereby fueling fruiting body expansion. The optimal 4 h daily GL photoperiod indicates that *T. giganteum* requires a defined light signal rather than continuous illumination, possibly to synchronize diurnal metabolic rhythms for efficient resource allocation (Table 4 and Table 5).

Beyond growth and yield, light quality significantly reshaped the physiological and nutritional profile, directly linking cultivation practice to postharvest quality. The elevation of SOD activity and DPPH scavenging capacity under RL (mycelium, Table 6) and GL (fruiting body, Table 7) suggests an induction of the antioxidant defense system [34]. Higher antioxidant capacity is directly correlated with extended shelf-life by delaying enzymatic browning, in which PPO plays a key role [18,35]. In support of this, GL-treated fruiting bodies maintained lower PPO activity alongside a lighter, more desirable milky-white color (Table 3 and Table 7), underscoring a tangible postharvest benefit.

Furthermore, the significant boost in polysaccharide and glutamate content under GL treatment (Table 10 and Table 11) underscores its role in enhancing functional and sensory quality. Glutamate is a primary umami compound [36], and its increased concentration directly contributes to improved flavor. Notably, the 4 h GL photoperiod not only maximized yield but also achieved the highest polysaccharide and glutamate contents (Table 11), demonstrating that moderate GL exposure synergistically optimizes both biomass and key quality metabolites. These GL-mediated improvements across multiple parameters—yield, morphology, color stability, and bioactive content—present a compelling, integrated case for its adoption in the fruiting phase.

In conclusion, we propose a two-stage lighting protocol: BL for rapid and robust mycelial colonization, followed by a 4 h daily GL photoperiod to maximize fruiting body yield, improve morphology, delay spoilage, and enrich nutritional value. This strategy moves beyond the empirical use of WL. A key limitation of this study is the descriptive level of mechanism; future work employing transcriptomics should target the identification of *T. giganteum* photoreceptors and the downstream genes regulating carbohydrate metabolism and secondary metabolite biosynthesis under different light spectra.

## 5. Conclusions

This study systematically investigated the effects of light quality on the growth and physiological characteristics of *T. giganteum*. The results indicated that BL significantly promoted mycelial growth and improved morphological development, whereas GL markedly enhanced the yield, agronomic traits, and key nutritional components of fruiting bodies. Furthermore, GL treatment was found to increase antioxidant enzyme activities, which contributed to delayed browning and better preservation of commercial quality. Based on these findings, we propose a stage-specific lighting strategy for industrial production: BL during the mycelial phase to stimulate growth and nutrient accumulation, followed by daily GL exposure (4 h) in the fruiting stage to synergistically optimize yield and quality. Although this study has clarified the regulatory effects of light quality on phenotypic and compositional traits, the underlying molecular mechanisms, particularly those related to light signal transduction and associated metabolic pathways, remain to be fully elucidated. Future studies integrating transcriptomic and metabolomic analyses are warranted to unravel the molecular basis of light-mediated development and secondary metabolism in *T. giganteum*. Such efforts would further refine photobiological theory and support more precise applications in the cultivation of edible fungi.

## Figures and Tables

**Table 1:** Mycelium growth of *T. giganteum* under different light qualities.

Light Quality	Illumination Time					
	3 d		5 d		10 d	
	Mycelial Traits	Growth Speed (mm·d^−1^)	Mycelial Traits	Growth Speed (mm·d^−1^)	Mycelial Traits	Growth Speed (mm·d^−1^)
UV	++	0.32 ± 0.02 b	+	0.21 ± 0.06 c	+	0.18 ± 0.07 c
GL	++++	0.62 ± 0.06 ab	+++	0.46 ± 0.09 b	+++	0.44 ± 0.07 b
BL	++++	0.74 ± 0.05 a	++++	0.74 ± 0.07 a	++++	0.58 ± 0.09 a
WL	+++	0.56 ± 0.02 ab	+++	0.40 ± 0.09 b	++++	0.42 ± 0.06 b
RL	+++	0.52 ± 0.08 ab	++++	0.42 ± 0.05 b	+++	0.35 ± 0.09 b
CK	+++	0.59 ± 0.05 ab	++++	0.51 ± 0.05 a	+++	0.55 ± 0.11 b

Ultraviolet (UV), green light (GL), blue light (BL), white light (WL), red light (RL), and darkness as control (CK); very thin (+), thin (++), thick (+++), and very thick (++++). Different lower-case letters indicate a significant difference at *p* < 0.05.

**Table 2:** Growth of fruiting bodies of *T. giganteum* under different light quality.

Light Quality	Incubation Time (d)	Primordium Formation Time (d)	First Harvesting Time (d)	First Level (kg)	Yield (kg)	Average Yield (kg)	Bioconversion Rate (%)
GL	40	18	39	3.75 ± 0.13 a	3.86 ± 0.09 a	0.32 ± 0.05 a	66.4
BL	41	19	41	3.09 ± 0.21 b	4.02 ± 0.22 a	0.34 ± 0.01 a	67.0
RL	45	25	48	2.04 ± 0.11 b	3.47 ± 0.07 b	0.29 ± 0.02 b	57.8
YL	46	24	47	1.31 ± 0.07 c	2.12 ± 0.1 c	0.18 ± 0.08 b	35.4
CK	45	24	47	2.71 ± 0.16 b	3.86 ± 0.18 b	0.33 ± 0.03 a	64.2

CK group was under white light; Yield: The total weight of mushrooms among the 12 mushroom bags; First Level: The total weight of first-level mushrooms among the 12 mushroom bags; Average Yield: The average weight of mushrooms in 12 mushroom bags. Yield values represent the mean of five replicates ± SD; different lower-case letters indicate a significant difference at *p* < 0.05.

**Table 3:** Agronomic traits of *T. giganteum* fruiting bodies under different light quality.

Light Quality	Mean Total Length (mm)	Pileus			Diameter of Stipe (mm)		Average Single Weight (g)
		Color	Mean Thickness (mm)	Mean Diameter (cm)	Upper	Lower	
GL	16.34 ± 0.97 b	Milky white	23.47 ± 0.28 ab	14.64 ± 1.33 a	23.89 ± 2.35 a	44.84 ± 4.32 b	105.92 ± 1.55 a
BL	12.11 ± 1.31 c	Milky white	25.47 ± 0.54 a	12.57 ± 1.85 ab	24.47 ± 1.39 a	45.68 ± 2.72 a	99.32 ± 2.81 ab
RL	19.69 ± 1.46 a	Light yellow	25.12 ± 0.78 a	10.22 ± 1.08 b	27.03 ± 3.64 a	48.68 ± 1.84 a	103.56 ± 11.20 a
YL	16.21 ± 2.37 b	Deep yellow	19.43 ± 0.75 b	5.71 ± 0.54 c	24.87 ± 3.49 a	47.29 ± 5.05 a	69.12 ± 7.13 c
CK	11.62 ± 0.85 c	Deep yellow	23.85 ± 0.72 ab	9.52 ± 0.81 b	23.98 ± 1.97 a	45.85 ± 4.87 a	91.29 ± 11.33 b

CK group was under white light; Yield values represent the mean of five replicates ± SD; different lower-case letters indicate a significant difference at *p* < 0.05.

**Table 4:** Effect of different light durations of GL on the growth rate and yield of *T. giganteum*.

Time (h)	Incubation Time (d)	Primordium Formation Time (d)	First Harvesting Time (d)	First Level (kg)	Yield (kg)	Average Yield (kg)	Bioconversion Rate (%)
2	40	18	39	4.07 ± 0.18 ab	4.97 ± 0.31 a	0.41 ± 0.05 a	82.6
4	36	18	37	5.35 ± 0.24 a	5.91 ± 0.21 a	0.49 ± 0.01 a	98.4
8	40	18	39	3.57 ± 0.23 b	4.94 ± 0.09 a	0.41 ± 0.05 a	82.2
12	40	18	39	4.09 ± 0.11 ab	4.85 ± 0.42 a	0.40 ± 0.01 a	80.8
24	40	18	39	3.99 ± 0.24 ab	4.65 ± 0.18 b	0.39 ± 0.09 b	77.4
CK	45	24	47	3.84 ± 0.36 ab	4.84 ± 0.48 a	0.40 ± 0.02 a	80.4

CK group was under white light; Yield: The total weight of mushrooms among the 12 mushroom bags; First Level: The total weight of first-level mushrooms among the 12 mushroom bags; Average Yield: The average weight of mushrooms in 12 mushroom bags. Yield values represent the mean of five replicates ± SD; different lower-case letters indicate a significant difference at *p* < 0.05.

**Table 5:** Agronomic traits of *T. giganteum* fruiting bodies under different light durations of GL.

Time (h)	Mean Total Length (cm)	Pileus			Diameter of Stipe/(mm)		Average Single Weight/(g)
		Color	Mean Thickness (mm)	Mean Diameter (cm)	Upper	Lower	
2	22.70 ± 0.53 ab	Milky white	23.76 ± 1.57 ab	11.48 ± 1.53 b	26.43 ± 1.20 a	39.88 ± 0.80 a	95.73 ± 1.59 b
4	24.61 ± 0.43 a	Milky white	25.01 ± 1.78 a	12.98 ± 1.17 a	29.54 ± 3.75 a	41.39 ± 2.26 a	102.40 ± 5.28 a
8	19.20 ± 0.82 bc	Light yellow	24.01 ± 0.61 ab	12.25 ± 1.16 ab	20.95 ± 1.39 b	28.61 ± 1.85 b	96.28 ± 5.06 ab
12	19.32 ± 0.33 bc	Deep yellow	22.16 ± 2.16 c	11.90 ± 1.89 b	20.03 ± 1.03 b	26.86 ± 1.86 bc	94.99 ± 5.18 b
24	12.09 ± 0.09 d	Deep yellow	21.55 ± 1.55 c	11.32 ± 1.32 b	22.00 ± 1.01 b	28.20 ± 1.80 b	91.16 ± 4.10 b
CK	17.82 ± 0.92 c	Deep yellow	23.02 ± 0.76 bc	12.08 ± 0.73 ab	20.85 ± 2.52 b	29.81 ± 6.58 b	91.70 ± 4.51 b

CK group was under white light; Yield values represent the mean of five replicates ± SD; different lower-case letters indicate a significant difference at *p* < 0.05.

**Table 6:** Effect of different light quality on physiological enzyme activity and antioxidant activity of *T. giganteum*.

Light Quality	Illumination Time								
	3 d			5 d			10 d		
	SOD (U·mL^−1^)	DPPH (%)	PPO (U·mL^−1^)	SOD (U·mL^−1^)	DPPH (%)	PPO (U·mL^−1^)	SOD (U·mL^−1^)	DPPH (%)	PPO (U·mL^−1^)
UV	164.23 ± 0.68 b	141.32 ± 0.35 b	164.23 ± 6.8 b	141.32 ± 5.35 b	135.54 ± 8.14 a	118.29 ± 5.68 b	121.36 ± 5.89 b	82.71 ± 1.12 b	133.36 ± 6.11 b
GL	188.51 ± 0.12 a	199.15 ± 0.43 b	188.51 ± 9.2 a	199.15 ± 9.43 b	122.08 ± 12.82 ab	161.94 ± 5.45 b	189.25 ± 5.49 b	93.21 ± 2.12 b	146.51 ± 6.46 a
BL	183.41 ± 0.89 a	204.51 ± 0.11 ab	183.41 ± 8.9 a	204.51 ± 9.11 ab	122.88 ± 14.12 ab	127.32 ± 5.89 b	194.56 ± 6.12 ab	76.16 ± 1.22 b	153.76 ± 5.12 a
WL	187.19 ± 0.46 a	212.24 ± 0.62 ab	187.19 ± 4.6 a	212.24 ± 9.62 ab	118.79 ± 12.82 b	154.09 ± 5.12 b	202.74 ± 10.68 ab	83.75 ± 3.42 b	148.96 ± 5.89 a
RL	184.95 ± 0.89 a	276.11 ± 0.13 a	184.95 ± 8.9 a	276.11 ± 10.13 a	123.55 ± 11.26 ab	240.20 ± 10.49 a	261.14 ± 10.89 a	121.82 ± 5.11 a	144.21 ± 6.89 a
CK	186.72 ± 0.49 a	231.23 ± 0.42 ab	186.72 ± 4.9 a	231.23 ± 10.42 ab	118.51 ± 10.95 b	130.95 ± 5.89 b	213.57 ± 10.46 ab	89.21 ± 2.26 b	153.20 ± 4.49 a

CK group was under dark; Yield values represent the mean of five replicates ± SD; different lower-case letters indicate a significant difference at *p* < 0.05.

**Table 7:** Effect of different light quality on physiological enzyme activity and antioxidant in fruiting bodies of *T. giganteum*.

Light Quality	Enzyme Activity and Antioxidant		
	SOD (U·mL^−1^)	DPPH (%)	PPO (U·mL^−1^)
GL	120.78 ± 5.58 b	148.01 ± 6.11 a	125.19 ± 5.46 a
BL	186.06 ± 6.03 a	132.13 ± 5.03 b	112.81 ± 4.20 c
RL	142.83 ± 5.19 ab	76.13 ± 1.07 b	114.87 ± 4.36 b
YL	166.96 ± 5.63 ab	53.09 ± 1.01 c	136.39 ± 5.10 a
CK	121.74 ± 2.98 b	106.08 ± 4.14 b	113.36 ± 5.23 bc

CK group was under white light; Yield values represent the mean of five replicates ± SD; different lower-case letters indicate a significant difference at *p* < 0.05.

**Table 8:** Enzyme activity of *T. giganteum* fruiting bodies under different light durations of GL.

Time (h)	Enzyme Activity and Antioxidant		
	SOD (U·mL^−1^)	DPPH (%)	PPO (U·mL^−1^)
2	133.33 ± 6.19 ab	78.11 ± 1.05 b	113.61 ± 2.11 c
4	168.76 ± 5.63 ab	133.11 ± 5.04 ab	116.67 ± 2.46 c
8	176.06 ± 6.03 a	133.12 ± 5.02 ab	134.31 ± 2.01 a
12	138.83 ± 4.19 ab	133.11 ± 5.01 ab	126.17 ± 2.35 b
24	119.58 ± 5.56 b	139.01 ± 4.21 a	127.18 ± 2.37 b
0	118.64 ± 2.68 b	109.08 ± 4.22 b	115.46 ± 2.22 c

CK group was under white light; Yield values represent the mean of five replicates ± SD; different lower-case letters indicate a significant difference at *p* < 0.05.

**Table 9:** Effect of different light quality on nutrient content in the mycelium of *T. giganteum*.

Light Quality	Illumination Time								
	3 d			5 d			10 d		
	Nutrient Content (g·100 g^−1^)			Nutrient Content (g·100 g^−1^)			Nutrient Content (g·100 g^−1^)		
	Protein	Polysaccharide	Glutamate	Protein	Polysaccharide	Glutamate	Protein	Polysaccharide	Glutamate
UV	0.33 ± 0.01 e	0.39 ± 0.01 b	0.90 ± 0.04 c	0.58 ± 0.02 d	0.52 ± 0.02 d	0.94 ± 0.02 e	0.52 ± 0.02 c	3.15 ± 0.11 c	0.92 ± 0.04 b
GL	0.66 ± 0.05 cd	0.54 ± 0.05 a	1.47 ± 0.06 ab	0.65 ± 0.04 cd	0.59 ± 0.04 d	1.31 ± 0.03 a	1.75 ± 0.09 b	5.45 ± 0.12 ab	1.11 ± 0.09 a
BL	0.51 ± 0.02 de	0.61 ± 0.02 a	1.63 ± 0.07 a	0.85 ± 0.02 ab	0.84 ± 0.02 ab	1.16 ± 0.06 c	1.62 ± 0.06 b	6.45 ± 0.11 a	0.92 ± 0.07 b
WL	0.95 ± 0.06 b	0.50 ± 0.02 a	1.67 ± 0.05 a	0.98 ± 0.09 ab	0.76 ± 0.06 bc	1.23 ± 0.03 b	2.19 ± 0.09 b	5.03 ± 0.19 abc	0.94 ± 0.02 b
RL	0.80 ± 0.05 bc	0.45 ± 0.01 b	1.36 ± 0.03 b	0.74 ± 0.05 bc	0.87 ± 0.05 ab	1.11 ± 0.02 d	0.62 ± 0.02 c	4.02 ± 0.14 bc	0.93 ± 0.02 b
CK	1.61 ± 0.06 a	0.57 ± 0.01 a	1.32 ± 0.03 b	1.73 ± 0.06 a	0.71 ± 0.04 bcd	1.11 ± 0.05 d	3.55 ± 0.11 a	5.73 ± 0.16 ab	0.94 ± 0.04 b

CK group was under dark; Yield values represent the mean of five replicates ± SD; different lower-case letters indicate a significant difference at *p* < 0.05.

**Table 10:** Effect of different light quality on nutrient content in the fruit body of *T. giganteum*.

Light Quality	Nutrient Content (g·100 g^−1^)		
	Protein	Polysaccharide	Glutamate
GL	33.42 ± 0.94 b	31.01 ± 0.82 a	4.39 ± 0.14 a
BL	30.54 ± 0.91 c	18.51 ± 0.83 abc	3.50 ± 0.13 b
RL	35.33 ± 0.87 a	20.49 ± 0.66 ab	3.15 ± 0.17 c
YL	35.92 ± 0.89 a	20.42 ± 0.64 abc	3.19 ± 0.12 bc
CK	33.64 ± 0.85 b	20.31 ± 0.57 abc	4.15 ± 0.116 a

CK group was underwhite light; Yield values represent the mean of five replicates ± SD; different lower-case letters indicate a significant difference at *p* < 0.05.

**Table 11:** Nutrient content of *T. giganteum* fruiting bodies under different light durations of GL.

Time (h)	Nutrient Content (g·100 g^−1^)		
	Protein	Polysaccharide	Glutamate
2	33.88 ± 0.84 ab	36.51 ± 0.81 ab	3.35 ± 0.12 d
4	35.80 ± 0.91 a	38.17 ± 0.93 a	5.70 ± 0.13 a
8	34.33 ± 0.83 ab	35.17 ± 0.89 ab	5.75 ± 0.13 a
12	34.15 ± 0.94 ab	33.02 ± 0.84 ab	4.66 ± 0.14 c
24	33.48 ± 0.83 b	30.87 ± 0.81 b	4.50 ± 0.13 c
CK	34.46 ± 0.91 a	30.86 ± 0.81 b	4.48 ± 0.13 c

CK: WL; Yield values represent the mean of five replicates ± SD; different lower-case letters indicate a significant difference at *p* < 0.05.

## Data Availability

The original contributions presented in this study are included in the article. Further inquiries can be directed to the corresponding author.
